# PI3Kγ Deficiency Suppresses Cutaneous Squamous Cell Carcinoma Formation by Modulating the Tumour Microenvironment Rather Than by Directly Regulating Keratinocyte Proliferation

**DOI:** 10.1111/exd.70219

**Published:** 2026-02-06

**Authors:** Aya Toyoshima, Natsuko Noguchi, Tomoko Suzuki, Takako Kuroki, Masami Kagaya, Fumino Oda, Michihiro Kono, Junko Sasaki, Takehiko Sasaki, Hidehisa Saeki, Shin‐Ichi Osada

**Affiliations:** ^1^ Department of Dermatology & Plastic Surgery Akita University Graduate School of Medicine Akita Japan; ^2^ Department of Dermatology Nippon Medical School Tokyo Japan; ^3^ Department of Biochemical Pathophysiology/Lipid Biology, Medical Research Institute, Institute of Integrated Research Institute of Science Tokyo Tokyo Japan

**Keywords:** chemical carcinogenesis, cutaneous squamous cell carcinoma, PI3Kγ, tumour microenvironment

## Abstract

Phosphatidylinositol‐3 kinase (PI3K) is a central regulator of cell proliferation, survival, metabolism, and migration via the downstream AKT/mTOR pathway. Although activating mutations in the catalytic subunit of PI3Kα (p110α) have been documented in various cancers, including cutaneous squamous cell carcinoma (cSCC), the role of PI3Kγ (p110γ), which is predominantly expressed in immune cells, remains poorly defined in cSCC. To elucidate the function of p110γ in cSCC development, we compared tumour formation in wild‐type and *p110γ‐*deficient mice using both a chemical carcinogenesis model and a syngeneic cSCC cell implantation model. While genetic deletion or pharmacological inhibition of PI3Kγ did not affect keratinocyte proliferation or migration in vitro, *p110γ‐*deficient mice exhibited significantly delayed tumour onset, reduced tumour burden, and suppressed growth of implanted cSCC tumours in vivo. Immunohistochemical analyses revealed that total CD4^+^ T cell infiltration was unchanged, whereas CD8^+^ cytotoxic T cell infiltration was markedly increased and FoxP3^+^ regulatory T cells were significantly reduced in tumours from p110γ‐deficient mice, resulting in a substantially elevated CD8^+^/FoxP3^+^ ratio. Immunoblot analyses of tumour lysates further demonstrated increased CD8 expression and enhanced NF‐κB p65 phosphorylation in p110γ‐deficient tumours. These results indicate that PI3Kγ contributes to cSCC development not by directly driving tumour cell proliferation but by shaping an immunosuppressive tumour microenvironment. Targeting PI3Kγ may therefore represent a promising immunotherapeutic strategy to enhance cytotoxic T‐cell–mediated antitumour immunity in cSCC.

**Abbreviations**
ANOVA: analysis of variance
BrdU: 5‐bromo‐2′‐deoxyuridine
cSCC: cutaneous squamous cell carcinoma
DMBA: 7,12‐dimethylbenz(a)anthracene
PI3K: phosphatidylinositol‐3 kinase
TPA: 12‐*O*‐tetradecanoylphorbol‐13‐acetate

## 1 Introduction

Cutaneous squamous cell carcinoma (cSCC) is the second most common form of skin cancer, following basal cell carcinoma [1]. It typically arises in sun‐exposed areas, with ultraviolet (UV) radiation playing a central role in its pathogenesis by inducing frequent somatic mutations [2, 3]. Approximately 80% of somatic mutations in cSCC exhibit UV‐signature changes, such as C>T and CC>TT transitions, although many of these are synonymous and do not alter protein function [2]. However, recurrent non‐synonymous mutations in key genes—TP53, CDKN2A, NOTCH1/2, KMT2C/D, and FAT1—have been identified and are believed to contribute significantly to tumour development [4, 5, 6, 7, 8].

A widely used experimental model for studying cSCC is the two‐stage chemical carcinogenesis model in mice [9, 10]. In this model, 7,12‐dimethylbenz[a]anthracene (DMBA) acts as the initiating carcinogen, followed by repeated applications of 12‐O‐tetradecanoylphorbol‐13‐acetate (TPA) as a tumour promoter. DMBA induces DNA mutations, while TPA promotes clonal expansion of the mutated cells into papillomas. This system recapitulates key steps in human cSCC progression, including H‐ras activation, Trp53 inactivation, and epithelial‐mesenchymal transition (EMT), and in some cases leads to spindle cell transformation [11].

DMBA preferentially induces A>T and G>T transversions. In murine cSCCs generated by this model, these mutations account for nearly 80% of all changes, constituting a characteristic DMBA‐induced mutational signature [9]. Frequently altered genes include H‐ras, Trp53, Notch1, Syne2, Kmt2c, Kmt2d, and Fat1, many of which overlap with mutations found in human cSCC, though H‐ras mutations are more prevalent in mice [12, 13]. This genetic similarity supports the relevance of the mouse model to human cSCC pathogenesis [14].

Phosphatidylinositol‐3 kinase (PI3K) is a key signalling molecule that regulates cellular proliferation, metabolism, survival, and migration via the downstream AKT/mTOR pathway [15]. Activating mutations in this pathway are among the most common genetic alterations in human cancers [16]. PI3K is classified into three classes, with class I further divided into IA (PI3Kα, β, δ) and IB (PI3Kγ) [17, 18]. Class IA isoforms are activated by receptor tyrosine kinases, while class IB is activated by G protein‐coupled receptors.

PI3K enzymes consist of a catalytic and a regulatory subunit. The catalytic subunits—p110α, p110β, p110γ, and p110δ—exhibit tissue‐specific expression and functions [17, 18]. Mutations in PIK3CA, which encodes p110α, are well documented in various cancers, and recent studies estimate that PIK3CA hotspot mutations occur in about 5% of cSCCs [5, 19, 20]. In contrast, the role of PI3Kγ in cSCC remains poorly understood.

PI3Kγ is predominantly expressed in immune cells. While mice lacking p110γ are viable, they display impaired neutrophil migration and altered immune responses [21, 22]. In macrophages, PI3Kγ acts as a molecular switch that modulates the balance between immunosuppressive and immunostimulatory states [23]. Inhibition of PI3Kγ in macrophages promotes a pro‐inflammatory phenotype by activating the NF‐κB pathway and inhibiting C/EBPβ, enhancing CD8+ T cell activity. Indeed, *p110γ‐*deficient mice implanted with lung, breast, or human papillomavirus‐positive head and neck squamous cell carcinoma cells exhibit increased CD8+ T cell responses and improved outcomes following checkpoint inhibitor therapy [23]. However, it remains unclear whether PI3Kγ influences de novo tumour formation (carcinogenesis) in addition to modulating tumour growth. In this study, we used a chemical carcinogenesis model to elucidate the role of PI3Kγ in the initiation and progression of cSCC.

## 2 Materials and Methods

### 2.1 Animals

*P110γ‐*deficient mice on a C57BL/6 background were previously generated as described [21]. Because C57BL/6 mice are relatively resistant to DMBA/TPA‐induced skin tumorigenesis [9, 24], *p110γ‐*deficient mice were backcrossed for more than 10 generations with FVB/N mice, a strain that exhibits high susceptibility to chemical carcinogenesis [25]. Wild‐type, heterozygous, and homozygous littermates were used in all experiments. Experimental procedures were approved by the Institutional Animal Care and Use Committees of Akita University School of Medicine (approval No. 26‐1‐84) and Nippon Medical School (approval No. 2022‐046) and conformed to institutional guidelines for animal welfare.

### 2.2 Chemical Carcinogenesis Protocol

Skin tumours were induced using a classical two‐stage chemical carcinogenesis protocol [26]. The dorsal skin of 7‐ to 8‐week‐old mice was shaved and topically treated once with 25 nmol of DMBA (Sigma‐Aldrich, MO, USA) dissolved in 200 μL of acetone (initiation). Beginning 1 week later, mice received biweekly topical applications of 6.8 nmol of TPA (Sigma‐Aldrich) in 200 μL acetone for a period of 6 months (promotion). Tumour development was monitored weekly. Tumours greater than 2 mm in diameter were counted, and animals were photographed at each time point. At the endpoint, tumour number and size were quantified prior to histological and molecular analyses.

### 2.3 Establishment of Syngeneic cSCC Cell Lines

Tumours from chemically induced cSCC were aseptically excised from donor FVB/N mice and minced using sterile scalpels. Tissue fragments were enzymatically dissociated in Dulbecco's modified Eagle medium (DMEM) supplemented with 0.5% collagenase type I (Thermo Fisher Scientific, MA, USA) and incubated at 37°C for 2 h with gentle agitation. After centrifugation and washing, the cell pellet was treated with 0.25% trypsin/0.02% EDTA (Thermo Fisher) for 10 min at 37°C. The resulting single‐cell suspension was passed through a 100‐μm cell strainer and seeded in HuMedia‐KG2 serum‐free keratinocyte medium (Kurabo, Osaka, Japan) containing defined growth factors and antibiotics. Primary cultures were expanded and characterised for epithelial morphology and epidermal markers before use in implantation experiments (Figure S1).

### 2.4 Histology and Immunohistochemistry

Mice were euthanized by cervical dislocation. For assessment of proliferation, 5‐bromo‐2′‐deoxyuridine (BrdU; Sigma‐Aldrich) was injected intraperitoneally at 100 μg/g body weight 3 h prior to sacrifice. Skin and tumour tissues were fixed overnight in 4% paraformaldehyde, dehydrated, and embedded in paraffin. Four‐micrometre sections underwent deparaffinisation and rehydration; antigen retrieval was performed using citrate buffer (pH 6.0) or Tris/EDTA buffer (pH 9.0) at 95°C for 20 min. Endogenous peroxidase activity was quenched with 3% hydrogen peroxide. Sections were blocked with normal serum and incubated overnight at 4°C with primary antibodies (antibody details in Table S1). Detection was achieved using the Vectastain Elite ABC Kit (Vector Laboratories, CA, USA), followed by colour development with the NovaRED substrate kit (Vector Laboratories). Slides were counterstained with haematoxylin and imaged using a BZ‐8000 microscope (KEYENCE, Osaka, Japan). CD4^+^, CD8^+^, and FoxP3^+^ cells were quantitated with BZ analyser software (KEYENCE).

### 2.5 Cell Viability Assay

To examine the effect of PI3Kγ inhibition on keratinocyte proliferation, primary keratinocytes were seeded at 1 × 10^5^ cells/well in 24‐well plates and treated with increasing concentrations (0–50 nM) of the selective PI3Kγ inhibitor AS252424 (Selleck Chemicals, TX, USA). AS252424 is a potent PI3Kγ inhibitor (IC_50_ = 30 nM) with 30‐fold selectivity over PI3Kα and minimal activity against PI3Kδ/β. Cell viability was assessed on Days 1 and 7 using alamarBlue reagent (Thermo Fisher). After incubation with 10% alamarBlue in phenol red–free DMEM for 4 h at 37°C, 100 μL of supernatant was transferred to a 96‐well plate, and fluorescence was measured at 540 nm excitation and 590 nm emission using a Fluoroskan Ascent Microplate Reader (Thermo Fisher). All experiments were performed in triplicate.

### 2.6 In Vitro Wound Healing Assay

Primary keratinocytes from wild‐type, heterozygous, and homozygous mice were seeded at 8 × 10^5^ cells per well onto type I collagen‐coated glass coverslips in 12‐well plates and cultured in HuMedia‐KG2 medium until confluence. A linear scratch was created using a sterile 200‐μL pipette tip, and medium was replaced with fresh serum‐free medium. Cells were incubated for 48 h at 37°C, fixed in methanol, and stained with 0.5% crystal violet (in 50% methanol). Wound closure was imaged and quantified using ImageJ software (National Institutes of Health, MD, USA) as previously described [27].

### 2.7 Tumour Implantation Experiments

To assess the role of PI3Kγ in the tumour microenvironment, we performed subcutaneous tumour implantation. Cultured syngeneic cSCC cells (1 × 10^6^ to 1 × 10^7^ cells) established as described above were resuspended in 100 μL of phosphate‐buffered saline and injected into the dorsal skin of wild‐type and *p110γ* ^−/−^ mice. Tumour growth was assessed every 3–4 days, and mice were sacrificed on Day 15 or Day 21. Tumours were weighed at the experimental endpoint after careful removal of the overlying skin, photographed, and processed for histological and molecular analyses.

### 2.8 Immunoblotting

Tumours were excised and immediately frozen in liquid nitrogen. Tissues (~25 mg) were homogenised in RIPA buffer (Fujifilm Wako, Osaka, Japan) supplemented with protease and phosphatase inhibitor cocktails. Lysates were cleared by centrifugation and quantified using a BCA protein assay (TaKaRa, Shiga, Japan). Equal amounts of protein (5–10 μg) were resolved by SDS‐PAGE on precast gels (Bio‐Rad, CA, USA) and transferred to PVDF membranes (Bio‐Rad). Membranes were blocked with 5% skim milk and probed overnight with primary antibodies (Table S1) followed by horseradish peroxidase‐conjugated secondary antibodies. Signals were visualised using the Clarity Western ECL kit (Bio‐Rad) and imaged on a ChemiDoc XRS system (Bio‐Rad).

### 2.9 Statistical Analysis

All quantitative data are presented as mean ± standard deviation (SD) or median, as indicated. A *p* value < 0.05 was considered statistically significant. All statistical analyses and graphical representations were generated using GraphPad Prism (version 10; GraphPad Software, San Diego, CA, USA).

## 3 Results

### 3.1 PI3Kγ Deficiency Markedly Suppresses Tumour Formation in a Chemical Carcinogenesis Model

**FIGURE 1:**
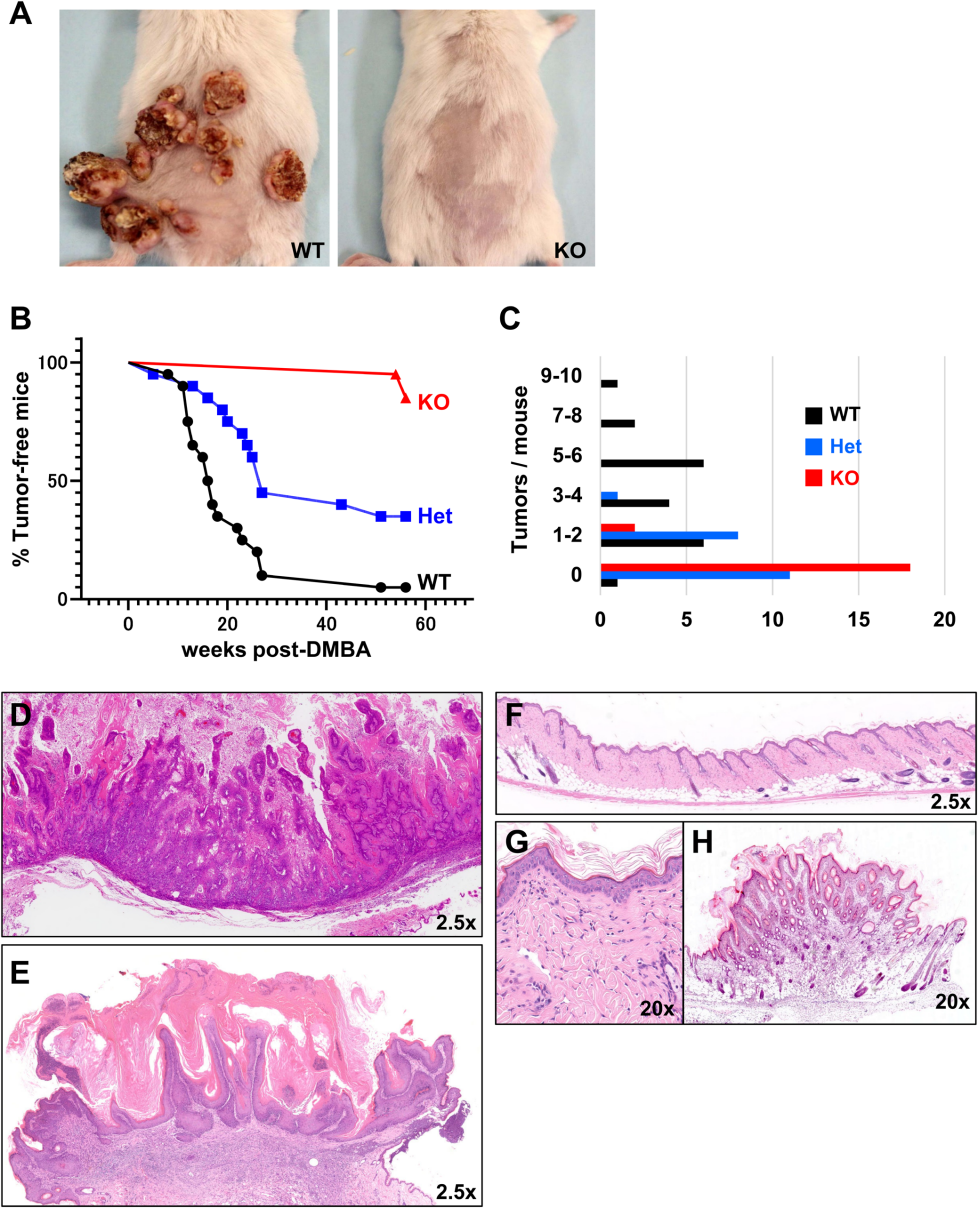
PI3Kγ deficiency suppresses tumour formation in a chemical carcinogenesis model. (A) Representative dorsal skin images of wild‐type (WT) and *p110γ* ^−/−^ homozygous knockout (KO) mice 6 months after DMBA/TPA treatment. Wild‐type mice developed multiple visible tumours, whereas tumour formation was minimal in KO mice. (B) Kaplan–Meier analysis of tumour‐free survival: Black line, WT (*n* = 20); blue line, *p110γ* ^*+/−*^ heterozygous (Het, *n* = 20); red line, KO mice (*n* = 20). Statistical significance was assessed using the log‐rank (Mantel–Cox) test (*p* < 0.0001). (C) Number of tumours per mouse. Tumour numbers were quantified at the experimental endpoint in WT (black line); Het (blue line); KO mice (red line). Tumour burden was significantly different among the three genotypes as assessed by the Kruskal–Wallis test (*p* < 0.0001). Post hoc pairwise comparisons using the Mann–Whitney *U* test revealed significant differences between WT and Het (*p* < 0.0001), WT and KO (*p* < 0.0001), and a modest but significant difference between Het and KO (*p* = 0.02). (D, E) Histopathological sections from wild‐type mice showing typical features of cSCC (D) and keratoacanthoma‐like cSCC (E). (F–H) Histopathological sections from KO mice showing either no epidermal abnormalities (F), mild hyperplasia (G), or benign papillomas (H), indicating protection from malignant transformation.

To investigate the role of PI3Kγ in cSCC development, we employed a two‐stage DMBA/TPA‐induced skin carcinogenesis model in wild‐type, *p110γ* ^+/−^ heterozygous, and *p110γ* ^−/−^ homozygous mice. Following 6 months of TPA treatment, wild‐type mice developed numerous tumours on the dorsal skin, whereas tumour development was drastically reduced in *p110γ* ^−/−^ mice (Figure 1A and Table S2). Tumours began to emerge in wild‐type and heterozygous mice around 10 weeks after DMBA application. The cumulative tumour incidence reached approximately 90% in wild‐type and 55% in heterozygous mice by Week 30. In contrast, only three of twenty *p110γ* ^−/−^ mice developed tumours, even after 56 weeks (Figure 1B), indicating a profound resistance to tumour formation in the absence of PI3Kγ. Kaplan–Meier analysis revealed a significant genotype‐dependent difference in tumour‐free survival, with *p110γ* ^−/−^ mice exhibiting a markedly delayed tumour onset compared with wild‐type and heterozygous mice (Figure 1B; log‐rank test, *p* < 0.0001).

The number of tumours per mouse was significantly reduced in *p110γ* ^−/−^ mice compared with wild‐type controls, while heterozygous mice exhibited an intermediate tumour burden (Figure 1C; *p* < 0.0001). Notably, wild‐type mice frequently developed multiple tumours, while most heterozygous animals developed only one or two. The few tumours observed in *p110γ* ^−/−^ mice were sparse and appeared later in the protocol. Histological examination revealed that tumours in wild‐type mice commonly displayed features of squamous cell carcinoma or keratoacanthoma‐like lesions (Figure 1D,E). In contrast, skin from *p110γ* ^−/−^ mice was either lesion‐free or contained only small benign papillomas with preserved epidermal architecture (Figure 1F–H), further supporting the protective effect of p110γ deletion against malignant progression.

### 3.2 TPA‐Induced Epidermal Proliferation Is Attenuated in *p110γ* ^−/−^ Mice

**FIGURE 2:**
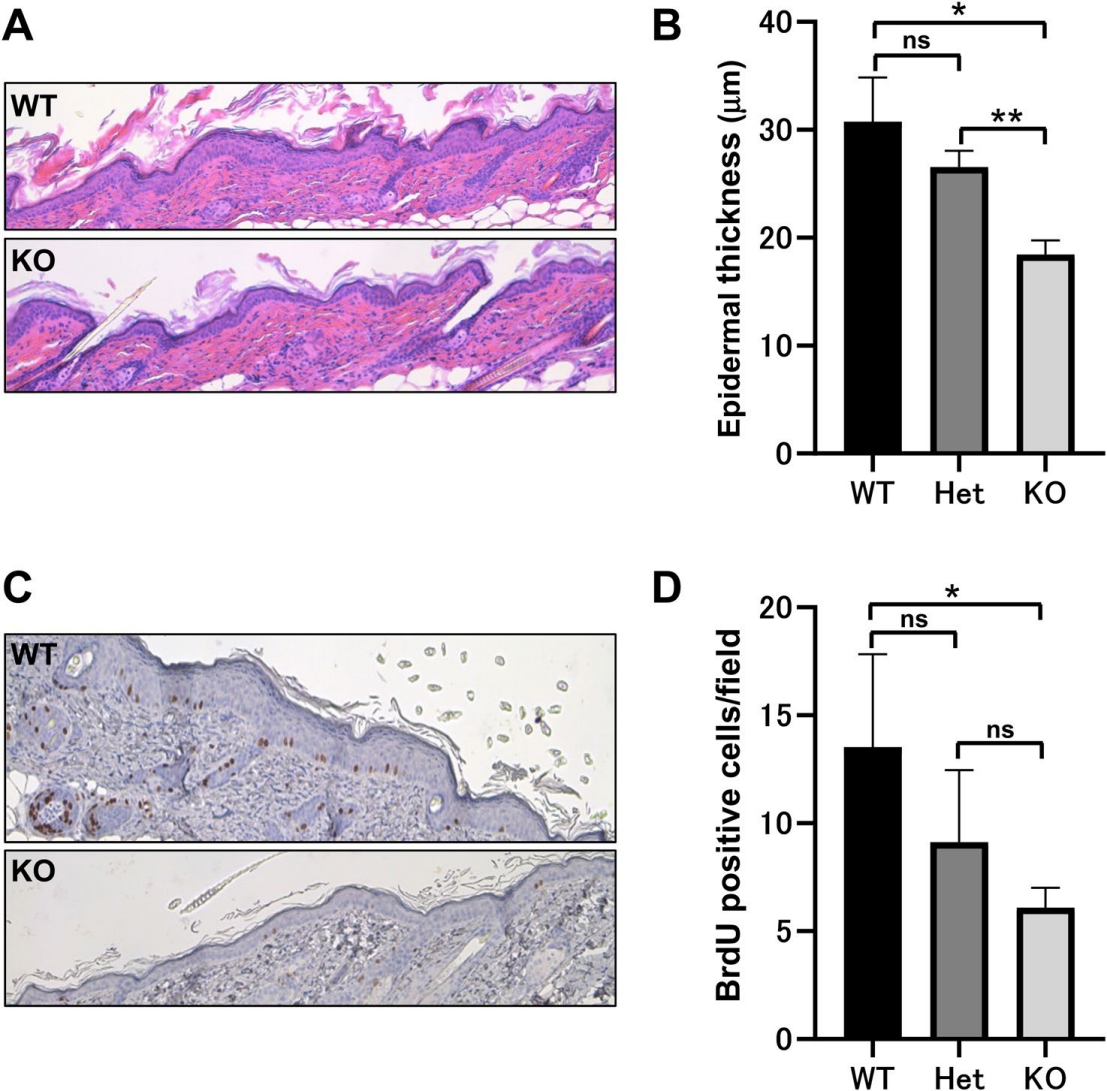
Epidermal proliferation is reduced in *p110γ‐*deficient mice following TPA application. (A) Representative haematoxylin and eosin–stained skin sections from WT and KO mice after a single application of DMBA followed by five applications of TPA. (B) Quantification of epidermal thickness in dorsal skin from WT, Het, and KO mice. The average thickness from the basal layer to the granular layer was measured at more than 20 randomly selected locations at 40× magnification per mouse. Data are presented as mean ± SD (*n* = 4 mice per group). Statistical significance was determined by Welch's one‐way ANOVA followed by post hoc tests assuming unequal variances. *p < 0.05; **p < 0.01; ns, not significant. (C) BrdU immunostaining of the epidermis of WT and KO mice. (D) Quantification of BrdU‐positive basal cell counts in WT (*n* = 3), Het (*n* = 4), KO (*n* = 4). Bars represent the mean ± SD of the number of BrdU‐positive cells per mouse (at least eight independent fields at 40× magnification). Statistical analysis was performed using the Kruskal–Wallis test followed by Dunn's multiple comparisons test. *p < 0.05; ns, not significant.

To assess whether the reduced tumorigenesis in *p110γ* ^−/−^ mice was related to altered keratinocyte proliferation, we analysed the dorsal skin following five applications of TPA. Histological examination revealed pronounced epidermal hyperplasia in wild‐type mice, whereas *p110γ* ^−/−^ mice exhibited minimal thickening (Figure 2A). Quantitative analysis confirmed a significant decrease in epidermal thickness in *p110γ* ^−/−^ animals compared with wild‐type controls (Figure 2B).

To further evaluate proliferative activity, BrdU incorporation assays were performed. Immunostaining showed fewer BrdU‐positive basal keratinocytes in *p110γ* ^−/−^ mice, indicating diminished proliferation in response to TPA (Figure 2C,D). These findings suggest that PI3Kγ contributes to early tumour promotion by supporting epidermal hyperproliferation in the inflammatory environment induced by TPA.

### 3.3 PI3Kγ Does Not Directly Regulate Keratinocyte Proliferation or Motility In Vitro

**FIGURE 3:**
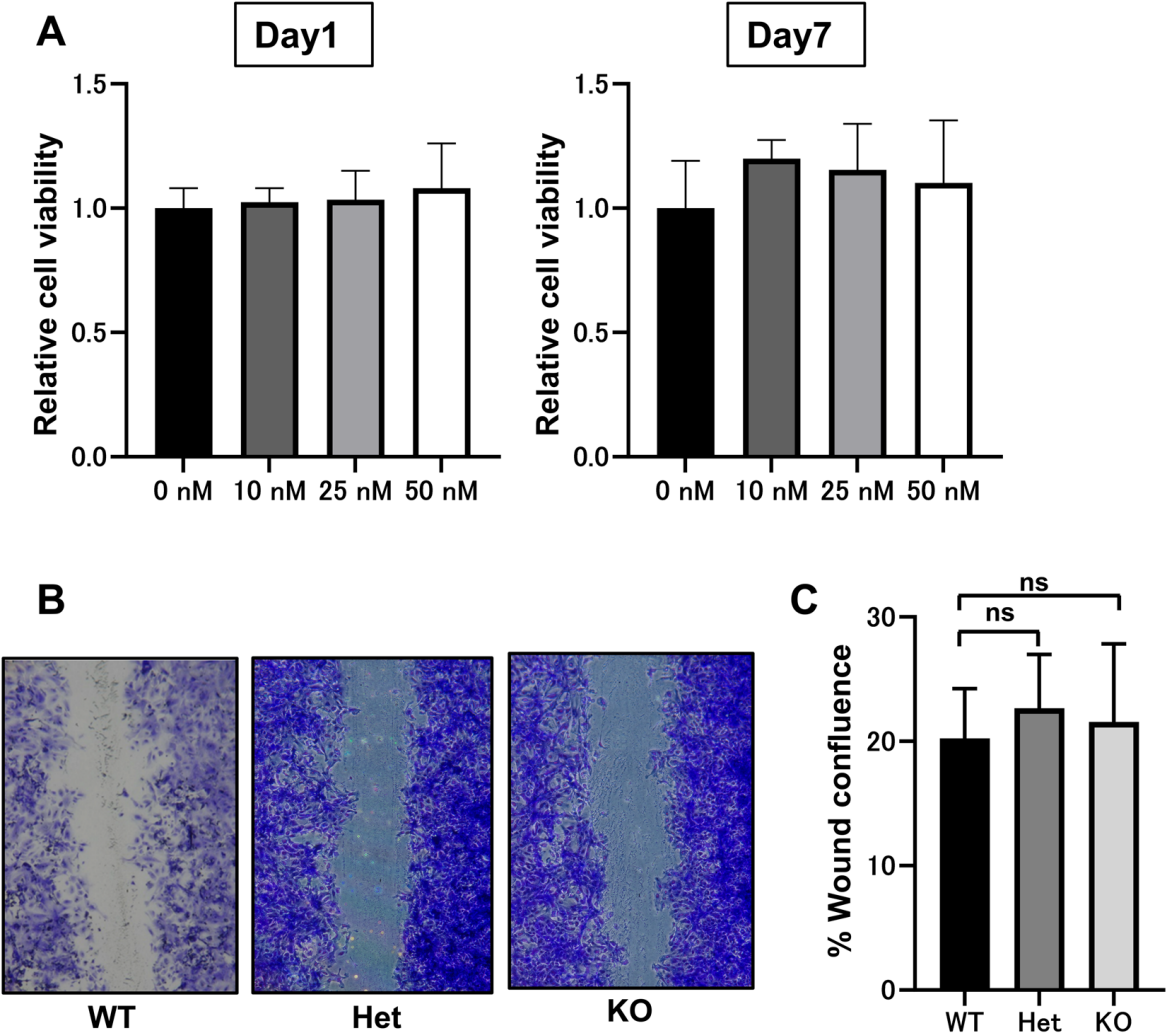
PI3Kγ inhibition does not affect keratinocyte proliferation or migration in vitro. (A) Cell viability after treatment with the indicated concentrations of AS252424 (a PI3Kγ‐specific inhibitor) for 1 and 7 days. Fluorescence values were normalised to the untreated control (0 nM) for each time point and are shown as mean ± SD (*n* = 3). Statistical analysis was performed using one‐way ANOVA with Dunnett's test versus control. (B) Scratch wound assay images at 48 h post‐wounding in WT, Het, and KO keratinocytes. (C) Gap closure at 48 h was quantified as the percentage of the remaining gap relative to 0 h. Data were analysed using the Kruskal–Wallis test followed by Dunn's multiple comparisons test. No statistically significant differences were detected among WT (*n* = 4), Het (*n* = 3), and KO (*n* = 5) groups.

Given that PI3Kγ is predominantly expressed in haematopoietic cells rather than keratinocytes, we next investigated whether PI3Kγ directly affects keratinocyte behaviour. Primary keratinocytes derived from wild‐type mice were cultured in the presence of AS252424, a selective PI3Kγ inhibitor. Cell viability, assessed over a 7‐day period, was not affected by the inhibitor even at concentrations up to 50 nM, which exceeded the IC_50_ value (Figure 3A), suggesting that PI3Kγ activity is dispensable for keratinocyte proliferation in vitro.

We also assessed keratinocyte migratory ability using a scratch assay. Wound closure rates over 48 h were comparable among keratinocytes derived from wild‐type, heterozygous, and *p110γ* ^−/−^ mice (Figure 3B,C). These results indicate that the reduced tumour formation and proliferation observed in vivo are unlikely to be due to cell‐autonomous effects of PI3Kγ in keratinocytes, supporting the hypothesis that PI3Kγ exerts its effects primarily through the tumour microenvironment.

### 3.4 Tumour Outgrowth of Implanted cSCC Cells Is Suppressed in *p110γ* ^−/−^ Mice

To further examine whether PI3Kγ modulates tumour progression through the host microenvironment, we performed tumour implantation experiments using syngeneic cSCC cells. The morphology of the cells was cobblestone‐like and immunoblotting was positive for epidermal markers: E‐cadherin, K14, and K6 (Figure S1).

**FIGURE 4:**
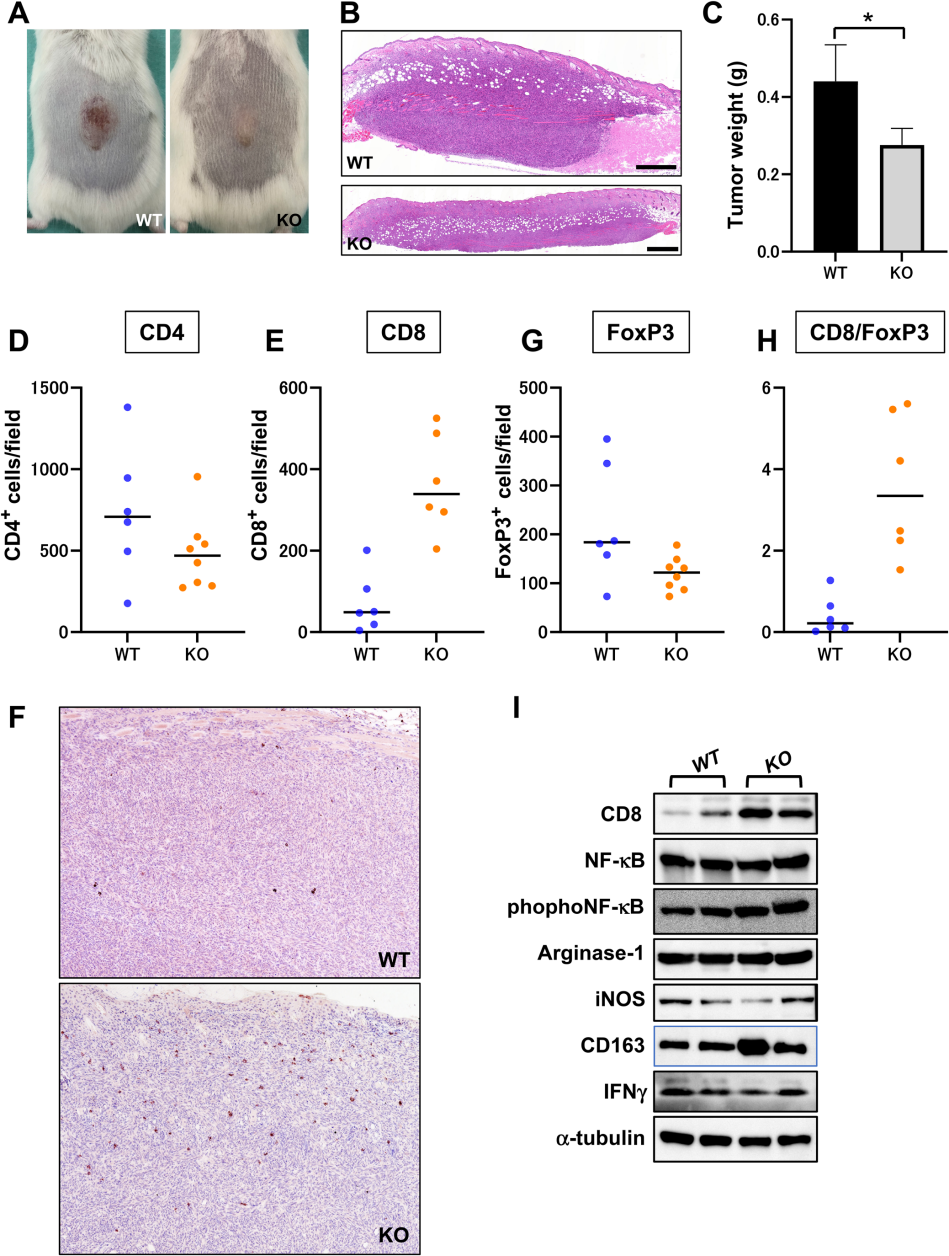
PI3Kγ deletion enhances CD8+ T cell infiltration and reshapes the immune microenvironment formed by implanted cSCC cells. (A) Gross appearance of subcutaneous tumours in WT and KO mice 21 days after tumour cell implantation. (B) Representative histopathological images of implanted tumours in WT and KO mice. Scale bar = 500 μm. (C) Final tumour weight measured at the experimental endpoint in WT (*n* = 4) and KO (*n* = 6) mice. Statistical significance was evaluated using a two‐tailed Mann–Whitney *U* test. *p < 0.05. Representative data from five independent experiments are shown. (D) Quantification of CD4^+^ T cells in tumour tissues from WT (*n* = 6) and KO (*n* = 8) mice (*p* = 0.27). (E) Quantification of CD8^+^ T cells in tumour tissues from WT (*n* = 6) and KO (*n* = 6) mice (*p* = 0.002). (F) Representative immunohistochemical staining for CD8 in tumour sections. Magnification, 40×. (G) Quantification of FoxP3^+^ regulatory T cells in tumour tissues from WT (*n* = 6) and KO (*n* = 8) mice (*p* < 0.05). (H) CD8^+^/FoxP3^+^ ratio calculated for each individual tumour (*p* = 0.002). (I) Immunoblot analysis of tumour lysates from WT and KO mice. α‐tubulin was used as a loading control. For panels D, E, G, and H, each dot represents one mouse. Bars indicate the median. Statistical significance was assessed using the Mann–Whitney *U* test.

Cells were subcutaneously injected into wild‐type and *p110γ* ^−/−^ mice, and tumour growth was monitored for up to 21 days (Table S3). While tumours grew steadily in wild‐type mice, tumour volume was significantly reduced in *p110γ* ^−/−^ mice (Figure 4A,B). At the endpoint, the average tumour weight was significantly reduced in *p110γ* ^−/−^ mice compared with wild‐type controls (Figure 4C; *p* = 0.028), demonstrating that PI3Kγ is also required for efficient tumour growth in established tumours.

### 3.5 Loss of PI3Kγ Enhances CD8+ T Cell Infiltration and NF‐κB Signalling in Tumours

To determine whether PI3Kγ deletion alters the immune composition of the tumour microenvironment, we focused on CD8^+^ cytotoxic T cells, total CD4^+^ T cells, FoxP3^+^ regulatory T cells, and the CD8^+^/FoxP3^+^ ratio as key indicators of antitumour immune balance [28, 29, 30].

Quantitative immunohistochemical analysis demonstrated that total CD4^+^ T cell infiltration was comparable between wild‐type and *p110γ* ^−/−^ tumours, with no statistically significant difference observed (Figure 4D; *p* = 0.27). In contrast, CD8^+^ T cell infiltration was significantly increased in *p110γ* ^−/−^ tumours relative to wild‐type tumours (Figure 4E; *p* = 0.002). Immunohistochemical staining for CD8 revealed a substantial increase in tumour‐infiltrating CD8+ T cells in *p110γ* ^−/−^ mice compared with wild‐type (Figure 4F). Moreover, FoxP3^+^ regulatory T cell infiltration was significantly reduced in *p110γ* ^−/−^ tumours compared with wild‐type tumours (Figure 4G; *p* < 0.05). Consistent with these findings, the CD8^+^/FoxP3^+^ ratio was markedly elevated in *p110γ* ^−/−^ tumours (Figure 4H; *p* = 0.002).

To further substantiate these findings, we performed immunoblotting using whole‐tumour lysates. Expression levels of CD8 were elevated in tumours from *p110γ* ^−/−^ mice, along with increased phosphorylation of NF‐κB p65 (Figure 4I), a key marker of immune activation. Although IFN‐γ was detectable, its expression levels were comparable between genotypes. Similarly, the expression of the canonical M1 marker iNOS and the M2 marker arginase‐1 did not show statistically significant differences. By contrast, expression of CD163 was increased in tumours from *p110γ* ^−/−^ mice. These findings indicate selective alterations in immune‐related protein expression in p110γ^−/−^ tumours.

## 4 Discussion

In this study, we identified PI3Kγ as a key regulator of cutaneous squamous cell carcinoma (cSCC) development in vivo. Using both chemical carcinogenesis and tumour implantation models, we demonstrated that genetic deletion of p110γ suppressed both tumour formation and progression. Importantly, our data indicate that PI3Kγ does not promote cSCC through direct effects on keratinocytes, but rather by shaping an immunosuppressive tumour microenvironment that restricts CD8+ T cell recruitment and function.

Previous studies have established a critical role for PI3Kγ in regulating innate and adaptive immunity [21, 22]. In macrophages, PI3Kγ acts as a molecular switch that promotes an immunosuppressive phenotype through activation of C/EBPβ and inhibition of NF‐κB signalling [23]. Conversely, genetic or pharmacological inhibition of PI3Kγ has been shown to induce an inflammatory transcriptional program and enhance antitumour immunity in models of breast, lung, and head and neck cancers [23]. However, these studies primarily focused on tumour growth and progression, leaving the role of PI3Kγ in tumour initiation largely unexplored.

Our findings extend these observations by demonstrating that PI3Kγ also promotes the initiation of epithelial tumours, as evidenced by the striking suppression of DMBA/TPA‐induced tumour formation in *p110γ* ^−/−^ mice. The early phase of tumour development in the chemical carcinogenesis model is characterised by TPA‐induced inflammation, proliferation of initiated keratinocytes, and clonal expansion [9]. We observed that TPA‐induced epidermal hyperplasia and BrdU incorporation were significantly reduced in *p110γ* ^−/−^ mice (Figure 2), indicating that PI3Kγ contributes to the tumour‐promoting environment in vivo. Importantly, in vitro experiments revealed that neither pharmacological inhibition nor genetic deletion of PI3Kγ affected keratinocyte proliferation or migration (Figure 3). These findings strongly support an indirect mechanism of tumour promotion mediated by the inflammatory and immune milieu rather than by direct effects on epithelial cells.

Consistent with this interpretation, PI3Kγ deficiency selectively altered the composition of tumour‐infiltrating immune cells. While total CD4^+^ T cell infiltration was not significantly affected (Figure 4D), p110γ^−/−^ tumours exhibited a marked reduction in FoxP3^+^ regulatory T cells, a key immunosuppressive subset of CD4^+^ T cells (Figure 4G), indicating a selective effect on immunosuppressive CD4^+^ T cell subsets. In parallel, we observed a robust increase in CD8^+^ T cell infiltration in p110γ^−/−^ tumours (Figure 4E,F), which represented the most prominent immunological change associated with PI3Kγ deficiency and likely contributed to the delayed tumour onset and reduced tumour burden observed in these mice. Immunohistochemical and immunoblot analyses further confirmed increased CD8^+^ tumour‐infiltrating lymphocytes and enhanced NF‐κB signalling within p110γ^−/−^ tumours (Figure 4I). These findings collectively support the concept that PI3Kγ maintains an immunosuppressive tumour microenvironment that limits effective antitumour T cell responses.

As a consequence of increased CD8^+^ T cell infiltration and reduced FoxP3^+^ regulatory T cell accumulation, the CD8^+^/FoxP3^+^ ratio was markedly elevated in p110γ^−/−^ tumours (Figure 4H). This ratio is widely regarded as an integrated indicator of antitumour immune competence, reflecting the balance between effector and immunosuppressive T cell populations [29, 30]. The elevated CD8^+^/FoxP3^+^ ratio therefore provides a mechanistic link between PI3Kγ deficiency and suppressed tumour development, reinforcing the notion that PI3Kγ promotes tumour progression by maintaining an immunosuppressive immune balance within the tumour microenvironment rather than by broadly enhancing lymphocyte infiltration.

Notably, the unchanged expression levels of the canonical M1 marker iNOS and the M2 marker arginase‐1 indicate that PI3Kγ deficiency does not simply alter macrophage abundance or classical M1/M2 polarisation. In this context, the increased expression of CD163, a macrophage scavenger receptor linked to tissue remodelling and inflammatory resolution [31], suggests a functional shift in macrophage states rather than enhanced immunosuppressive polarisation. Together, these findings support the notion that PI3Kγ deficiency reshapes the tumour immune microenvironment toward a state permissive for CD8^+^ T cell–mediated antitumour immunity without broadly altering macrophage quantity or canonical polarisation status.

A limitation of this study is that the immunoblot analyses were performed using bulk tumour lysates, which contain heterogeneous cell populations including macrophages, myeloid‐derived suppressor cells, fibroblasts, and tumour cells. Consequently, the expression levels of M1/M2‐associated proteins such as iNOS and arginase‐1 may not accurately reflect macrophage subset–specific activity. Future cell‐type–specific approaches, including flow cytometry and single‐cell transcriptional profiling, will be required to precisely define the immune subsets regulated by PI3Kγ signalling.

Although IFN‐γ was detectable in tumour lysates, we did not observe a robust quantitative difference between genotypes (Figure 4I), suggesting that PI3Kγ deficiency primarily affects immune cell composition and activation state rather than inducing large alterations in bulk cytokine levels. Additionally, PI3Kγ may influence tumour vasculature or lymphatic architecture. Recent studies have highlighted the role of high endothelial venules (HEVs) in facilitating lymphocyte entry into tumours and shaping responses to immunotherapy [32]. Whether PI3Kγ regulates HEV formation, vascular permeability, or adhesion molecule expression represents an important topic for future investigation.

Beyond mechanistic insights, our findings have important therapeutic implications. cSCC is a highly prevalent skin cancer with rising incidence, particularly among elderly and immunosuppressed individuals. Notably, the high tumour mutational burden of cSCC, largely resulting from chronic ultraviolet (UV) exposure, renders this malignancy particularly amenable to immune checkpoint inhibition [33]. Although immune checkpoint inhibitors (ICIs) have demonstrated efficacy in advanced cSCC [34], a substantial proportion of patients fail to respond and experience immune‐related adverse events [35, 36]. While responsiveness to ICIs was not directly evaluated in the present study, our data demonstrate that PI3Kγ inhibition enhances CD8^+^ T cell–mediated antitumour immunity and reshapes the tumour immune microenvironment toward a less immunosuppressive state, which may provide a rationale for future studies exploring combination strategies with immune checkpoint blockade. In line with this concept, PI3Kγ‐selective inhibitors such as eganelisib (IPI‐549) have demonstrated synergy with ICIs in preclinical studies and are currently under clinical investigation [37, 38, 39]. Accordingly, a combinatorial approach incorporating PI3Kγ inhibition with immune checkpoint blockade—potentially at reduced ICI doses—could be explored as a strategy to maintain antitumour efficacy while mitigating immune‐related toxicity [40], which may be particularly advantageous for elderly patients or those with pre‐existing autoimmune comorbidities.

In conclusion, our study demonstrates that PI3Kγ promotes both the initiation and progression of cSCC by maintaining an immunosuppressive tumour microenvironment. Genetic deletion of PI3Kγ reprograms the immune landscape toward enhanced CD8^+^ T cell infiltration and NF‐κB–mediated immune activation, thereby unleashing effective antitumour immune responses. These findings provide a strong rationale for targeting PI3Kγ as a novel immunomodulatory strategy for both the treatment and prevention of cSCC.

## Author Contributions

**Aya Toyoshima:** investigation, visualisation, writing‐original draft. **Natsuko Noguchi:** investigation, visualisation. **Tomoko Suzuki:** investigation. **Takako Kuroki:** investigation. **Masami Kagaya:** investigation. **Fumino Oda:** investigation. **Michihiro Kono:** reviewing and editing. **Junko Sasaki:** methodology, resources, reviewing and editing. **Takehiko Sasaki:** methodology, resources, reviewing and editing. **Hidehisa Saeki:** funding acquisition, writing‐reviewing. **Shin‐Ichi Osada:** conceptualization, investigation, supervision, funding acquisition, writing‐reviewing and editing.

## Funding

This study was supported by the Japan Society for the Promotion of Science (JSPS) Grant‐in‐Aid for Scientific Research (C): H.S. (#20K08698) and S.‐I.O. (#23K07752).

## Conflicts of Interest

The authors declare no conflicts of interest.

## Supporting information


**Table S1:** Lists the antibodies used for immunoblotting and immunohistochemistry.

**Table S2:** Summarises the results of the chemical carcinogenesis experiments.

**Table S3:** Summarises the tumour cell implantation experiments.

**Figure S1:** Shows the morphological and immunological characterisation of syngeneic cSCC cells.

## Data Availability

The data that supports the findings of this study are available in the Supporting Information of this article.
