# Supplementary material for: PI3Kγ Deficiency Suppresses Cutaneous Squamous Cell Carcinoma Formation by Modulating the Tumour Microenvironment Rather Than by Directly Regulating Keratinocyte Proliferation
Source: Exp Dermatol. 2026 Feb 6;35(2):e70219. doi: 10.1111/exd.70219 (PMC12880963; doi:10.1111/exd.70219)
Supplement: Supplementary file 4 — Figure S1: Shows the morphological and immunological characterisation of syngeneic cSCC cells. [file EXD-35-e70219-s002.zip › exd70219-sup-0004-FigureS1.docx]

**Legend for Supplementary Figure**

**Supplementary Figure 1.**

(A) Morphology of syngeneic cSCC cells used for tumor implantation, confirming epithelial characteristics. Bar = 50 μm. (B) Immunoblot analysis of epidermal protein expression in syngeneic cSCC cells. Lane 1: E-cadherin (135 kDa); Lane 2: Keratin 14 (50 kDa); Lane 3: Keratin 6 (60 kDa). Molecular weight markers (kDa) are shown on the left.
