# Supplementary figures and images for: PI3Kγ Deficiency Suppresses Cutaneous Squamous Cell Carcinoma Formation by Modulating the Tumour Microenvironment Rather Than by Directly Regulating Keratinocyte Proliferation
Source: Exp Dermatol. 2026 Feb 6;35(2):e70219. doi: 10.1111/exd.70219 (PMC12880963; doi:10.1111/exd.70219)

## Slide 1
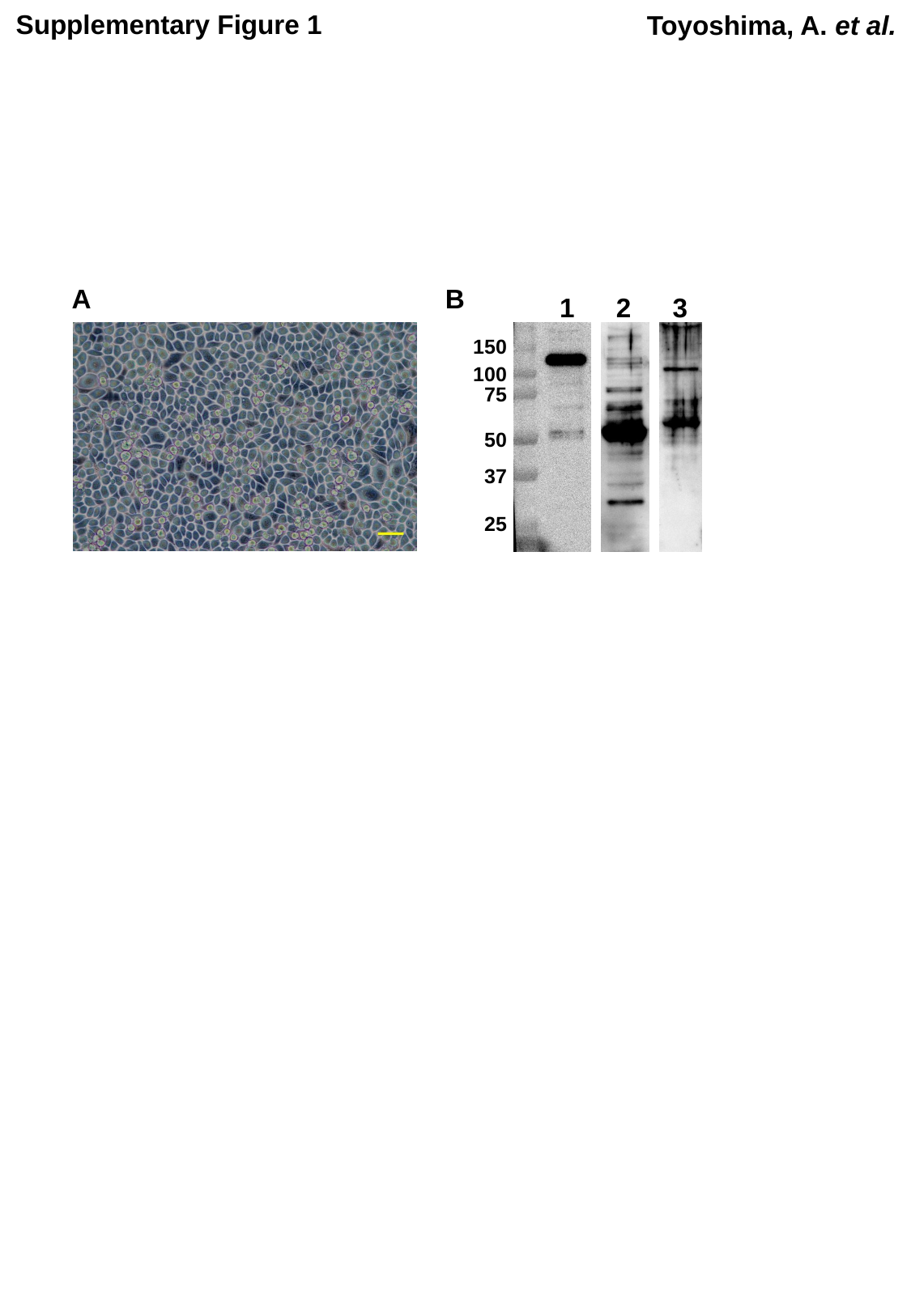

Supplementary Figure 1
Toyoshima, A. et al.
A
B
1
2
3
150
100
75
50
37
25

Supplement: Supplementary file 4 — Figure S1: Shows the morphological and immunological characterisation of syngeneic cSCC cells. [file EXD-35-e70219-s002.zip › exd70219-sup-0005-FigureS1@Supplementary Figure1.pptx]
